# Identification of Leaf Waxy Candidate Gene and Expression Changes in Related Genes in Response to Cold Stress of Cabbage (*Brassica oleracea* L.)

**DOI:** 10.3390/cimb48020152

**Published:** 2026-01-30

**Authors:** Dengkui Shao, Yanjing Ren, Changrong Deng, Junqin Wen, Baohua Li, Quanhui Li, Lugang Zhang

**Affiliations:** 1 State Key Laboratory for Crop Stress Resistance and High-Efficiency Production, College of Horticulture, Northwest A&F University, Yangling 712100, China; 2 Academy of Agriculture and Forestry Sciences, Qinghai University, Xining 810016, China; 3 Laboratory of Research and Utilization of Germplasm Resources in Qinghai-Tibet Plateau, Qinghai University, Xining 810016, China; 4 Qinghai Key Laboratory of Vegetable Genetics and Physiology, Xining 810016, China

**Keywords:** waxy-cabbage, wax accumulation, fine mapping, *BoCER1*, α-linolenic acid metabolic, cold stress, regulatory pathway

## Abstract

In cabbage, epidermal wax plays a key role in adaptation to abiotic and biotic stresses. The glossy green cabbage variety, which has less wax, is becoming increasingly popular on the market. In this study, the highly inbred waxy cabbage HQ2-1 and the glossy green cabbage Y2-1 were sampled for fine mapping and transcriptomics analysis. In the glossy green leaf cabbage, inheritance follows a simple dominant pattern. BSA-seq and interval targeted sequencing technology identified *BoCER1* as the candidate gene controlling the leaf wax trait in *Brassica oleracea*. Downregulated genes in the α-linolenic acid metabolic pathway and upregulated genes in the wax synthesis pathway in HQ2-1 collectively promote wax formation in HQ2-1 leaves. Cold stress induced the upregulation of α-linolenic acid metabolic pathway genes in HQ2-1, and we speculate that the upregulation of these genes may promote jasmonic acid accumulation. Our study lays a solid foundation for further understanding the regulatory mechanism of leaf wax formation in cabbage and for the translational application of breeding new glossy cabbage varieties.

## 1. Introduction

Cabbage (*Brassica oleracea* L. var. *capitata*) is an important staple biennial vegetable belonging to the Brassicaceae family, characterized by its leafy green, red, or white foliage. Having been widely cultivated for over 4000 years, it remains one of the most productive and economically valuable crops among Brassicaceae vegetables. Cabbage is rich in vitamin C, K, B6, dietary fiber, and glucosinolates, which possess antioxidant, anti-inflammatory, and anticancer activities [1]. As one of the top ten vegetable crops worldwide, more than 70 million tons of cabbage is produced annually, supporting multiple industrial chains, including fresh consumption, processing (such as the production of kimchi and dehydrated vegetables), and livestock feed. Furthermore, crop rotation systems including cabbage positively contribute to improving soil structure and mitigating continuous cropping obstacles [2].

The epidermal wax on cabbage is an important component of its cuticle, playing a key role in the plant’s adaptation to abiotic and biotic stresses by reducing water evaporation, reflecting ultraviolet radiation, and resisting pathogen invasion [3]. The main components of epidermal wax are long-chain fatty acids, aldehydes, ketones, esters, alkanes, and other hydrophobic compounds [4]. The composition and content of epidermal wax are influenced by both genetic and environmental factors, such as light and temperature, with significant variation differences observed among different varieties [5].

Many genes important to the biosynthesis of cabbage wax have been identified. Aarts et al. [6] found that the *cer1-1* mutant is characterized by a drastic decrease in products of the alkane-forming pathway (alkanes, secondary alcohols, and ketones) and a corresponding increase in aldehydes. Bourdenx et al. [7] reported that overexpression of *CER1* promotes biosynthesis of very-long-chain alkanes. Pascal et al. [8] found that *CER1* and *CER3* can produce waxy alkanes through an aldehyde decarboxylation reaction, and their expression levels are significantly positively correlated with cuticular hydrophobicity. Wang et al. [9] identified that *KCS1*, *KCS2*, and *KCS6* regulate the carbon chain elongation of very-long-chain fatty acids (VLCFAs) and exhibit higher expression levels in wax alkane synthesis. Schnurr et al. [10] reported that *LACS2* is the rate-limiting enzyme responsible for activating fatty acids in wax ester synthesis. Using CRISPR/Cas9 technology, Cao et al. [11] successfully knocked out the *KCS* and *CER* genes, significantly reducing wax content and verifying their function. Yang et al. [12] discovered that overexpression of *CER1* improves drought tolerance in kale by regulating cuticular wax biosynthesis. Song et al. [13] reported that reducing the wax content can enhance leaf tenderness, thereby satisfying the requirements of fresh consumption markets. Although many genes involved in leaf wax formation have been identified, the differentially expressed structural genes in the key metabolic pathway of leaf wax formation have not been systematically investigated.

A series of studies have reported that waxes support resistance to low-temperature stress in different types of plants. Ladaniya MS [14] reported that wax-coated mandarin fruit has a longer shelf life when stored at a chilling temperature with intermittent warming. Zhu et al. [15] reported that in tea plants, n-hexadecanoic acid is positively related to cold resistance. Darré et al. [16] found that wax treatment enhances tolerance to chilling injury in red bell pepper. Bourdenx et al. [7] reported that overexpression of CER1 enhances resistance to biotic and abiotic stresses. Our previous research also found that waxy cabbage seedlings are more tolerant to low temperatures [17]. However, how CER1 mediates the response of waxy cabbages to low temperatures via altering lipid metabolism remains unclear.

In this study, we analyzed the genetic characteristics of waxy leaf cabbages, identifying *BoCER1* as the candidate gene for the waxy leaf phenotype in HQ2-1. We further examined the gene expression changes involved in wax formation on cabbage leaves and predicted the conversion of α-linolenic acid to jasmonic acid under cold stress. Our study lays a solid foundation for understanding the regulatory mechanism of leaf wax formation in cabbage.

## 2. Materials and Methods

### 2.1. Plant Materials

The highly inbred line of waxy leaf cabbage HQ2-1 and the glossy green cabbage Y2-1 were used as parents to generate F_1_-1, F_1_-2, BC_1_-1, BC_1_-2, and F_2_ populations for inheritance studies, genetic mapping, and transcriptomic analysis to identify differentially expressed genes. The F_1_-1 population was created by crossing HQ2-1 with Y2-1, and the BC_1_-1 population was generated by crossing individual F_1_-1 plants with HQ2-1. The F_1_-2 population was created by crossing Y2-1 with HQ2-1, and the BC_1_-2 population was generated by crossing individual F_1_-2 plants with Y2-1. The F_1_-1 and F_1_-2 populations were each used to generate F_2_ populations separately. Healthy seeds from all individuals were sown in 72-hole plates filled with seedling substrate, and the plates were placed in a sunlit greenhouse. When the seedlings reached the four-leaf-one-heart stage, they were transplanted into the field at Qinghai University, where they were managed using normal water and fertilizer until the cabbage heads became firm. All plant materials used in this study were provided by the Vegetable Breeding Team, Institute of Horticulture, Academy of Agriculture and Forestry Sciences, Qinghai University.

### 2.2. Genetic Characteristics of Wax Traits of Cabbage Leaves

The wax phenotypes of F_1_-1, F_1_-2, BC_1_-1, BC_1_-2, and F_2_ individuals were investigated when the cabbage seedlings reached the three-leaf stage and rosette stage, separately. The numbers of waxy and wax-free plants in the F_1_ and F_2_ generations, respectively, were counted, and the segregation ratio was calculated.

### 2.3. Fine Mapping

Thirty individual plants with thick wax and thirty individual wax-free plants from the F_2_ population were selected to construct the waxy and wax-free pools, respectively. Samples from the HQ2-1, Y2-1, and waxy and wax-free pools were subjected to re-sequencing and BSA-sequencing by Beijing Biomarker Tech Co., Ltd. (Beijing, China), following the instructions of Wang et al. [18].

Based on the initial positioning results, polymorphic SNP markers with PolyHighRes sequencing data were converted into KASP markers. The KASP primers were designed using the PolyMarker website (http://www.polymarker.info/, accessed on 29 December 2025), and all primers were synthesized by Baimaike Co., Ltd. (Beijing, China). For each SNP locus, two forward primers and one reverse universal primer were designed and named Primer-A, Primer-B, and Primer-C, respectively. Primer-A and Primer-B represented the two alleles of the SNP, with FAM and HEX fluorescent labels added. Genotype analysis of the two parent samples was performed using Primer Mix to screen for polymorphic KASP primers. Subsequently, the genotype of the F_2_ population was analyzed using these polymorphic KASP markers. Fluorescence data from the PCR reaction were read using a microplate reader, and genotype data at different loci were analyzed using KlusterCaller software (version 4.1). Interval-targeted sequencing technology was then employed to further sequence the 904 kb candidate interval.

### 2.4. Candidate Gene Prediction and Cloning

The physical positions of flanking markers linked to leaf waxy traits were determined using the BLAST (version 2.17.0+) network online service, and candidate gene prediction was performed with the GBrowse tool from BRAD (http://brassicadb.cn/#/BLAST/, accessed on 29 December 2025). Specific primers for the candidate gene were designed based on the reference genome of *B. oleracea* [19]. The candidate gene sequences were cloned from HQ2-1. PCR products were purified using a TaKaRa MiniBEST agarose Gel DNA Extraction Kit (TaKaRa, Shiga, Japan) and used for TA cloning. The purified PCR products were inserted into the pMD19-T Simple Vector (TaKaRa, Japan) and transformed into Escherichia coli strain DH5α. Recombinant plasmids were sequenced by Aoke Biotechnology Co., Ltd. (Yangling, China), and sequence alignment was conducted using DANMAN software (version 10.0.2.100). The function of the predicted gene was searched using the BLASTP tool (version 2.17.0+) from NCBI.

### 2.5. Functional Marker Development

According to the re-sequencing results, a large fragment deletion occurred in the candidate gene region of HQ2-1 and Y2-1. Therefore, a specific primer pair was designed and synthesized to amplify a functional marker, *BoCER1*. PCR amplifications were performed to detect *CER1* variations in the two parent cabbage plants and F_2_ individuals.

### 2.6. Transcriptome Analysis of HQ2-1 and Y2-1 Leaves

Healthy HQ2-1 and Y2-1 seeds were sown in 72-hole plates filled with seedling substrate, and the plates were placed in a sunlit greenhouse. When the seedlings reached the four-leaf-one-heart stage, their leaves were collected for transcriptome analysis. Three biological replicates were set for each sample, with each replicate consisting of eight individual plants. RNA extraction, library construction, library quality control, RNA sequencing, sequencing data quality control, and reference genome comparison were all performed by Wuhan Genoseq Technology Co., Ltd. (Wuhan, China). An Illumina HiSeq Paired-end 150 bp platform (Illumina, San Diego, CA, USA) was used for sequencing. Correlation analysis and principal component analysis (PCA) were also conducted. DESeq2 v1.10.1 software was employed to identify differentially expressed genes using the following criteria: |log2 fold change| ≥ 1 and a false discovery rate (FDR) < 0.05.

### 2.7. Stress Treatment

Healthy waxy cabbage HQ2-1 seedlings with uniform growth at the four-leaf-one-heart stage were subjected to stress treatment. For PEG-simulated drought stress, the cabbage seedlings were immersed in a 25% PEG-6000 solution and placed in a light incubator at 22 °C/15 °C with 70% humidity [20,21]. For cold stress, the seedlings were transferred to a light incubator at 4 °C with 70% humidity [22]. For heat stress, they were moved to a light incubator at 35 °C with 70% humidity [23]. To simulate salt–alkali stress, the seedlings were immersed in a 150 mmol/L NaHCO_3_ solution and placed in a light incubator at 22 °C/15 °C with 70% humidity. The treatment durations were 48, 96, and 144 h.

### 2.8. Determination of Free Fatty Acids and Data Analysis

Cabbage leaf samples weighing 0.1 g were collected in a centrifuge tube, and a free fatty acid (FFA) content detection kit (Shanghai Liquid Chromatography-Mass Spectrometry Detection Technology Co., Ltd., Shanghai, China) was used to determine their free fatty acid content. There were three replicates for each sample, with each replicate containing 5 individual plants. Microsoft Excel 2019 was used for data organization, and Origin 2021 software was used to plot graphs.

### 2.9. Low-Temperature Treatment for Transcriptome Analysis

Healthy HQ2-1 and Y2-1 seeds were sown in 72-hole plates filled with seedling substrate, and the plates were placed in a sunlit greenhouse. When the seedlings reached the three-leaf-one-heart stage, they were transferred to an artificial climate chamber for low-temperature stress treatment. The treatment conditions were set as follows: day/night temperatures of 10 °C/5 °C, day/night photoperiods of 14 h/10 h with a light intensity of 12,000 Lx, 60% air relative humidity, and treatment maintained for 7 days. The control group had day/night temperatures of 20 °C/10 °C, with all other conditions being consistent. Three biological replicates were established for each sample, with each replicate containing 8 individual plants. Differentially expressed gene identification analysis was performed following the methods used in the transcriptome analysis of HQ2-1 and Y2-1 leaves.

## 3. Results

### 3.1. Genetic Characteristics of Waxy Leaf Cabbages

**Figure 1:**
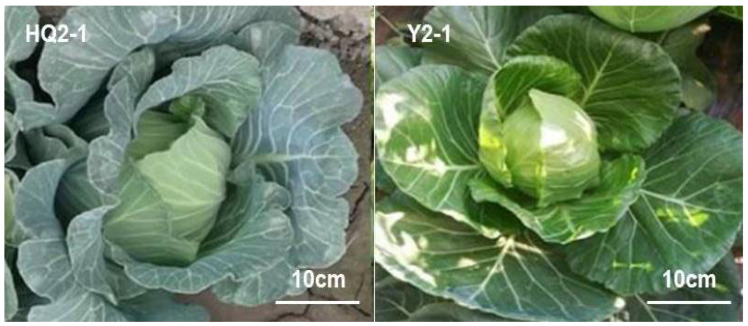
Genetic characteristics of wax deficiency trait in parental materials.

The highly inbred waxy leaf cabbage HQ2-1 (P1) and the glossy green cabbage Y2-1 (P2) (Figure 1) were used as parents to generate F_1_-1 and F_1_-2 cabbages. The wax phenotype of F_1_-1, F_1_-2, BC_1_-1, BC_1_-2 and F_2_ individuals was investigated when the cabbage seedlings reached the three-leaf stage and rosette stage, separately. Single plants with consistent wax phenotype identification were used for subsequent studies. All F_1_-1 (P1 × P2) and F_1_-2 (P2 × P1) individuals were found to be wax-free, indicating that the wax-free trait is dominant, without cytoplasmic effects. Due to the similarity in phenotype between F_1_-1 and F_1_-2 individuals, F_2_ populations derived from F_1_-1 and F_1_-2 cabbages were combined when calculating the separation ratio. In the F_2_ separation population, the ratio of wax-free to waxy plants was 1191:384, which is approximately 3.102:1. Chi-square testing confirmed that this ratio fits a 3:1 distribution (χ^2^ = 0.072, χ^2^_0.05_ = 3.841), suggesting that the wax-free trait in the Y2-1 cabbage is controlled by a single dominant gene (Table 1).

In the BC_1_-1 [(P1 × P2) × P1] separation population, an obvious segregation ratio of 1:1 was observed, indicating that the genotype of BC_1_-1 individuals was heterozygous dominant and homozygous recessive as Waxwax and waxwax. In the BC_1_-2 [(P1 × P2) × P2] population, all individuals were found to be wax-free, indicating that the genotype of BC_1_-2 individuals was homozygous dominant and heterozygous dominant as WaxWax and Waxwax (Table 1). All F_1_-1 (P1 × P2) and F_1_-2 (P2 × P1) individuals were heterozygous dominant (Waxwax). Thus, we inferred that the waxy phenotype in HQ2-1 (P1) was homozygous recessive (waxwax) and the wax-free phenotype in Y2-1 (P2) was homozygous dominant (WaxWax).

### 3.2. Fine Mapping of Candidate Genes for Wax Traits in Cabbage Leaves and Identification of Candidate Genes

**Figure 2:**
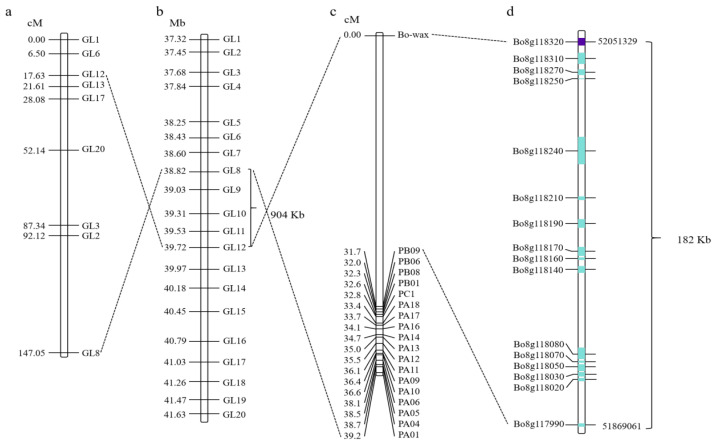
Fine mapping of candidate genes responsible for wax traits of cabbage leaves. (**a**,**b**) The wax formation gene was initially mapped within the GL8-GL12 interval on chromosome C08. (**c**,**d**) The candidate gene for wax formation was mapped to a region of 182 kb using interval targeted sequencing technology. The numbers on the left side of the bars in (**a**–**c**) represent genetic distances. The line connecting the two diagrams indicates that this is the same molecular marker. The length of the blue box indicates the gene length, while the purple box denotes the candidate gene.

Based on re-sequencing of parental DNA and BSA pool sequencing, the gene responsible for wax formation on the surface of cabbage leaves was initially located within the GL8-GL12 interval on chromosome C08. Subsequently, Competitive Allele-Specific PCR (KASP) markers were used to further map the candidate gene for wax formation. Through collinearity analysis, the candidate gene region was narrowed to a 904 kb range (Figure 2a,b). Interval-targeted sequencing technology was used to further sequence the 904 kb candidate interval; finally, the candidate gene for wax formation was located in a 182 kb region (Figure 2c,d). A total of 16 annotated genes were identified in this 182 kb region (Supplementary Table S1). Based on the gene annotation of the *B. oleracea* reference genome, three of these sixteen genes may be candidates, including *Bo8g117990* (Sec14p-like phosphatidylinositol transfer family protein gene), *Bo8g118020* (Phosphatidylinositol N-acetylglucosaminyltransferase subunit P-like protein gene), and *Bo8g118320* (CER1 gene associated with stem epicuticular wax production and pollen fertility). Furthermore, based on high-throughput targeted sequencing data, the read coverage of the target gene region in HQ2-1 and Y2-1 revealed that *Bo8g118320* in Y2-1 had a deletion of the 3′ end DNA fragment. Therefore, we hypothesized that *Bo8g118320* is the candidate gene controlling leaf wax formation. Gene function annotation indicated that *Bo8g118320* encodes an aldehyde decarboxylase involved in cutin, suberin, and wax biosynthesis within the lipid metabolic pathway, which is also known as *CER1*.

### 3.3. Candidate Gene Clones and Molecular Marker Design

Based on the *B. oleracea* reference genome, the gDNA and CDS of *Bo8g118320* from HQ2-1 were cloned, with the full-length gDNA being 3516 bp and the CDS being 2055 bp. Genetic structure analysis revealed that the gene contains 10 exons and 9 introns. Due to a large fragment deletion of *Bo8g118320* in Y2-1, we were unable to obtain complete gDNA and CDS sequences for *Bo8g118320* in Y2-1. An analysis of physicochemical properties indicated that Bo8g118320 encodes an aldehyde decarboxylase with 684 amino acids, which is a stable protein. Analysis of the signal peptide and transmembrane structure domain revealed that the aldehyde decarboxylase polypeptide chain lacks a signal peptide but contains five transmembrane helical structure regions.

Based on the sequence of HQ2-1 and the high-throughput targeted sequencing results for Y2-1, a functional dominant molecular marker, F-R2, was designed. When this marker was used to amplify bands in waxy parents and waxy individual plants, bands were detected, but no bands were found in wax-free parents and wax-free individual plants (Figure S1).

### 3.4. Identification of Differentially Expressed Genes Involved in Wax Synthesis in HQ2-1 and Y2-1

To further explore the molecular mechanism underlying changes in leaf wax formation-related genes in cabbage, transcriptome analysis was performed using leaves from the waxy cabbage HQ2-1 and the wax-free cabbage Y2-1. Based on six samples, with an average of 11.45 Gb per sample, a total of 68.73 Gb of clean data were obtained, for which the mean Q30 value was 93.30%. The reads obtained from sequencing were aligned to the *Brassica oleracea* reference genome (v2.1.25), with an average alignment rate of 88.40%, in which 84.30% of the reads could be uniquely mapped to the reference genome (Supplementary Table S2). Correlation analysis of biological replicates in RNA-Seq experiments revealed that three replicates under identical treatments clustered together, while materials treated at different temperatures showed significant dispersion, confirming the high reproducibility of the test samples and marked differences among treatments (Figure S2).

**Figure 3:**
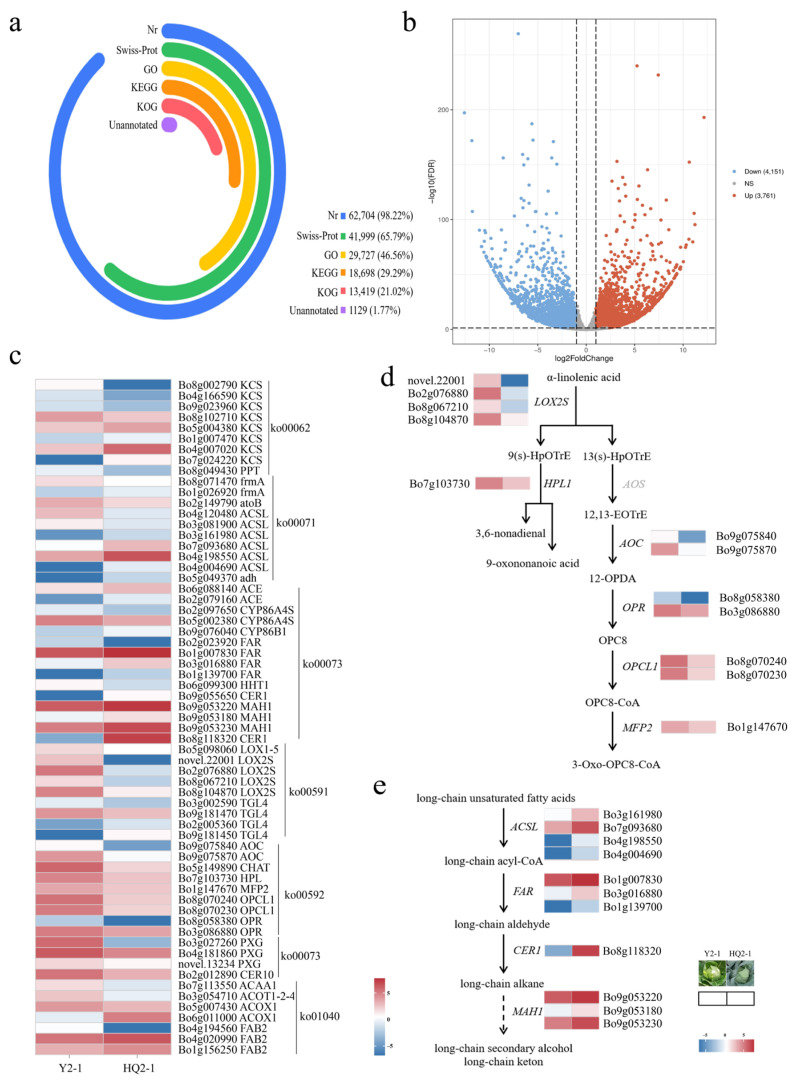
Identification of differentially expressed genes involved in wax synthesis in HQ2-1 and Y2-1. (**a**) Gene sequence annotation. (**b**) Volcano plot of differentially expressed genes between HQ2-1 and Y2-1. (**c**) Heatmap of differentially expressed genes involved in wax synthesis. (**d**) Reconstructed linolenic acid metabolic pathway based on the differentially expressed genes. (**e**) Reconstructed wax biosynthesis pathway based on the differentially expressed genes. Note: The gene expression heatmaps in (**d**,**e**) were derived from (**c**). 9(s)-HpOTrE: 9-hydroxy-octadecatrienoic acid. 13(s)-HpOTrE: 13-hydroxy-octadecatrienoic acid. 12,13-EoTrE: 12,13-Epoxyoctadeca-9,11,15-trienoic acid. 12-OPDA: 12-Oxophyto-10,15-dienoate. OPC8: 8-[(1S,2S)-3-Oxo-2-[(Z)-pent-2-enyl]cyclopentyl] octanoic acid.

Gene sequences were compared with those in KEGG, NR, Swiss-Prot, GO, KOG, and other databases, with annotation rates of 35.27% (7103 genes), 94.49% (19,029 genes), 65.95% (13,281 genes), 43.56% (8772 genes), and 28.14% (5668 genes), respectively, while 5.49% (1105 genes) remained unannotated (Figure 3a). RNA-Seq results showed that a total of 7912 genes were identified as differentially expressed genes. Among these, 3761 genes were upregulated and 4151 genes were downregulated at HQ2-1 (Figure 3b, Supplementary Table S3). Using the KEGG database, six pathways related to wax formation were identified, including cutin, suberine and wax biosynthesis (ko00073), alpha-linolenic acid metabolism (ko00592), linoleic acid metabolism (ko00591), biosynthesis of unsaturated fatty acids (ko01040), fatty acid elongation (ko00062), and fatty acid degradation (ko00071). In a comparison of HCK and YCK, a total of 63 differentially expressed genes were found in these six pathways, among which 26 were significantly upregulated and 37 were significantly downregulated in HCK (Figure 3c, Supplementary Table S4).

Based on the differentially expressed genes, the waxy formation pathway was reconstructed. In the linolenic acid metabolic pathway, α-linolenic acid enters two pathways under the catalysis of different enzymes. Four *LOX2S* genes (*Bo2g076880*, *Bo8g067210*, *Bo8g104870*, and *novel.22001*), which encode lipoxygenase and catalyze the conversion of α-linolenic acid into 9-hydroxy-octadecatrienoic acid (9(s)-HpOTrE) and 13-hydroxy-octadecatrienoic acid (13(s)-HpOTrE), were significantly downregulated in HCK. The *HPL* gene, which encodes linolenate hydroperoxidelyase and catalyzes the conversion of 9(s)-HpOTrE into 9-oxononanoic acid and 3,6-nonadienal, was also significantly downregulated in HCK. Meanwhile, the *AOS* gene, which encodes hydroperoxide dehydratase and catalyzes the conversion of 13(s)-HpOTrE into 12,13-Epoxyoctadeca-9,11,15-trienoic acid (12,13-EoTrE), showed no significance in the comparison between HCK and YCK. Two copies of *AOC* genes (*Bo9g075840* and *Bo9g075870*) encoding allene oxide cyclase, two copies of *OPR* genes (*Bo8g058380* and *Bo3g086880*) encoding 12-oxophytodienoic acid reductase, two copies of *OPCL1* genes (*Bo8g070240* and *Bo8g070230*) encoding OPC-8:0 CoA ligase 1, and the *MFP2* gene (*Bo1g147670*) encoding enoyl-CoA hydratase were all significantly downregulated in HCK (Figure 3d).

In the wax biosynthesis pathway, long-chain unsaturated fatty acids are converted into long-chain acyl-CoA under the catalysis of long-chain acyl-CoA synthetases. In the comparison of HCK vs. YCK, four *ACSL* genes (*Bo3g161980*, *Bo7g093680*, *Bo4g198550*, and *Bo4g004690*), which encode long-chain acyl-CoA synthetases, were significantly upregulated in HCK. Under the catalysis of acyl-CoA reductase, long-chain acyl-CoA is converted into long-chain aldehyde. Three *FAR* genes (*Bo1g007830*, *Bo3g016880*, and *Bo1g139700*), which encode acyl-CoA reductase, were significantly upregulated in HCK. The long-chain aldehyde was converted into a long-chain alkane under the catalysis of aldehyde decarbonylase. The *CER1* gene encoding aldehyde decarbonylase was significantly upregulated in HCK but nearly absent in YCK. The long-chain alkane was converted into a long-chain secondary alcohol and long-chain ketone under the catalysis of midchain alkane hydroxylase, and three copies of the *MAH1* genes (*Bo9g053220*, *Bo9g053180*, and *Bo9g053230*) encoding midchain alkane hydroxylase were significantly upregulated in HCK (Figure 3e).

Expression of the *CER1* gene, which encodes aldehyde decarbonylase, was directly blocked in YCK. We hypothesized that down-regulated expression of *CER1* in YCK may inhibit the conversion of long-chain aldehydes to long-chain alkanes, thereby suppressing the formation of long-chain alkanes, long-chain secondary alcohols, long-chain ketones, and long-chain diketones.

### 3.5. Leaf FFA Response to Stress in the Waxy Cabbage HQ2-1

**Figure 4:**
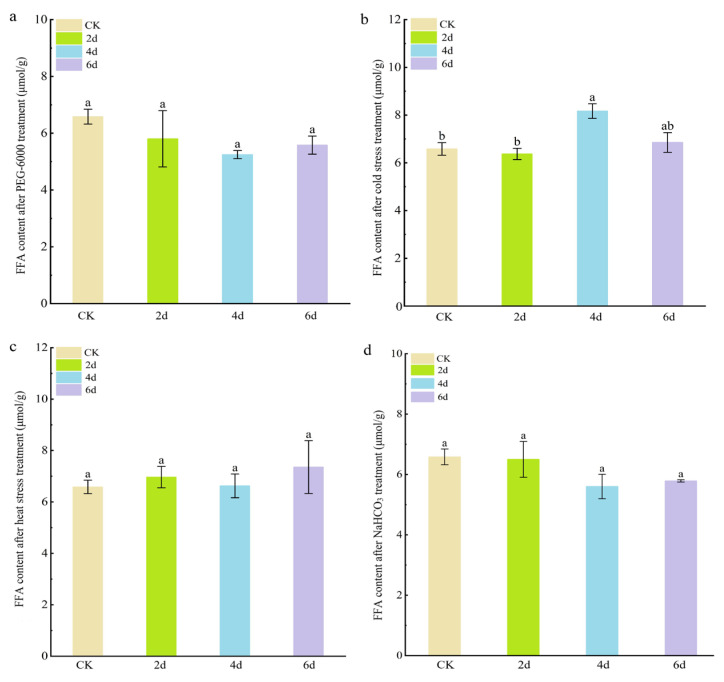
Free fatty acid content of cabbage seedlings after adverse stress treatment. (**a**) PEG-6000 simulated drought stress treatment. (**b**) Cold stress treatment. (**c**) Heat stress treatment. (**d**) NaHCO_3_ stress treatment. The letters above the columns indicate significance at *p* < 0.05.

Free fatty acids (FFAs) are the main components of leaf wax, so we investigated changes in FFA content to measure changes in leaf wax. The results showed that the FFA content in cabbage leaves did not change significantly after PEG-6000 simulated drought stress, heat stress, or NaHCO_3_ stress (Figure 4). However, under cold stress treatment, the FFA content in cabbage leaves was significantly higher after 4 and 6 days (8.17 ± 0.30 μmol g^−1^ FW and 6.85 ± 0.42 μmol g^−1^ FW) compared to the control (6.58 ± 0.45 μmol g^−1^ FW). These results suggest that cold stress can significantly promote the biosynthesis of free fatty acids.

### 3.6. Cold Stress Induced Upregulated Expression of α-Linolenic Acid Metabolic Pathway

**Figure 5:**
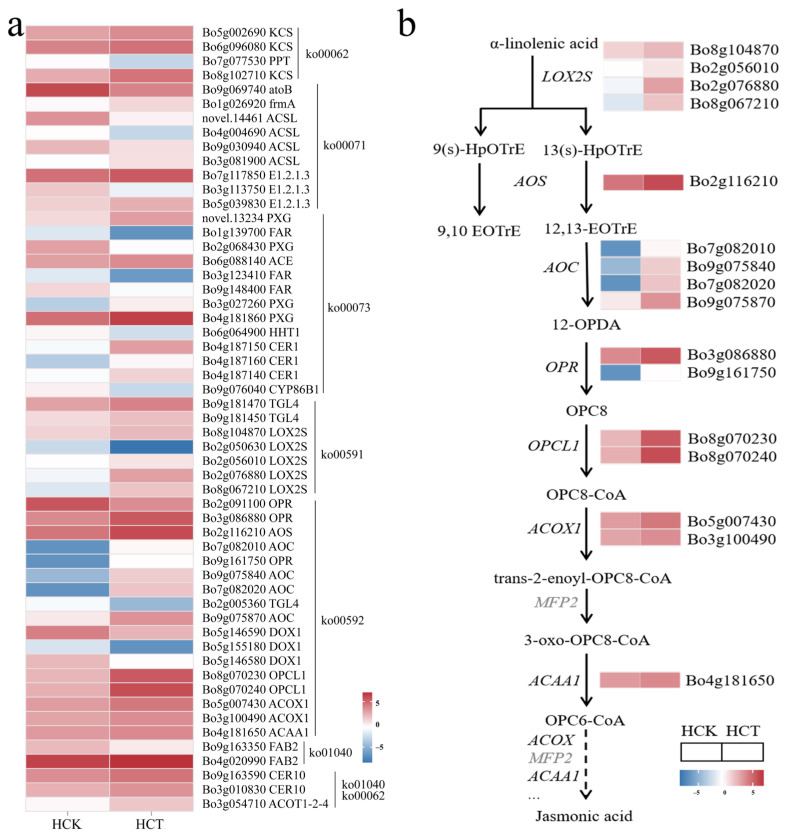
Differentially expressed gene analysis of HQ2-1 under cold stress. (**a**) Heatmap of differentially expressed genes in HCT vs. HCK. (**b**) Reconstructed linolenic acid metabolic pathway based on differentially expressed genes. Note: Heatmaps of gene expression in (**b**) were derived from (**a**). 9(s)-HpOTrE: 9-hydroxy-octadecatrienoic acid. 13(s)-HpOTrE: 13-hydroxy-octadecatrienoic acid. 12-OPDA: 12-Oxophytodienoate. 12,13-EoTrE: 12,13-Epoxyoctadeca-9,11,15-trienoic acid. OPC8: [(1S,2S)-3-Oxo-2-[(Z)-pent-2-enyl]cyclopentyl] octanoic acid.

To clarify the regulatory network of genes related to wax synthesis in response to cold stress, we used transcriptome sequencing to analyze differentially expressed genes in the waxy cabbage HQ2-1 under cold stress (HCT). A total of 8173 differentially expressed genes were identified, with 4129 upregulated and 4044 downregulated at HCT (Supplementary Table S5). Based on the six pathways related to wax formation, a total of 55 differentially expressed genes involved in wax synthesis were identified under cold stress, including 35 significantly upregulated genes and 20 significantly downregulated genes (Figure 5a, Supplementary Table S6).

Among these 55 differentially expressed genes, 17 were enriched in the linolenic acid metabolic pathway. Thus, we reconstructed the linolenic acid metabolic pathway under cold stress in HQ2-1 leaves. Four *LOX2S* genes (*Bo8g104870*, *Bo2g056010*, *Bo8g067210* and *Bo2g076880*), one *AOS* gene (*Bo2g116210*), four *AOC* genes (*Bo7g082010*, *Bo9g075840*, *Bo7g082020* and *Bo9g075870*), two OPR genes (*Bo3g086880* and *Bo9g161750*), and two *OPCL1* genes (*Bo8g070230* and *Bo8g070240*) were all significantly upregulated in HCT. The synergistic action of these genes converts α-linolenic acid into OPC8-CoA. Upregulated expression of these genes in HCT might promote the accumulation of OPC8-CoA. OPC8-CoA was then converted into isojasmonic acid CoA through the catalytic action of a series of enzymes, including acyl-CoA oxidase, enoyl-CoA hydratase, and acetyl-CoA acyltransferase. Two *ACOOX1* genes (*Bo3g100490* and *Bo5g007430*), encoding acyl-CoA oxidase, and the *ACAA1* gene (*Bo4g181650*), encoding acetyl-CoA acyltransferase, were all significantly upregulated in HCT. In contrast, the *MFP2* gene, which encodes enoyl-CoA hydratase, showed no significant difference when comparing HCT and HCK (Figure 5b).

Based on the gene expression level and the reconstructed linolenic acid metabolic pathway, we suspected that the upregulated genes in HCT promote the metabolic pathways of α-linolenic acid, thereby promoting the accumulation of jasmonic acid on the surface of HQ2-1 leaves under cold stress. The upregulated expression of genes in the α-linolenic acid metabolic pathway of HQ2-1 under cold stress also indirectly confirms that cold stress can significantly promote fatty acid biosynthesis.

## 4. Discussion

### 4.1. Wax Biosynthesis Candidate Genes in Brassica Crops

Many genes controlling wax synthesis in *Brassica* crops have been identified. In cabbage, Liu et al. [24] and Dong et al. [25] identified one candidate gene, *Bol018504*, which confers the glossy trait and is a homolog of *CER1* in *Arabidopsis thaliana*. Liu et al. [26] found that a 2722 bp insertion in the first intron of *Bol018504* occurs in the glossy mutant. However, Dong et al. [25] found no difference in the *Bol018504* sequence between CGL-3 and the wild type (WT). Liu et al. [26] reported that a single recessive locus, *BoWax1*, controls the glossy green trait. Using genetic mapping, Han et al. [27] identified a candidate gene, *BoGL5*, that is homologous to *Arabidopsis CER2*, which is essential for epicuticular wax biosynthesis in broccoli. Wang et al. [28] identified a single recessive gene, *Bol026949*, that controls the glossy green trait in cabbage and found that *Bol026949* may participate in cuticular wax production by regulating the transcript levels of genes involved in post-translational cellular processes and phytohormone signaling.

In this study, we identified a dominant single gene, *Bo8g118320*, that confers the glossy green trait in cabbage. This gene is a homolog of *CER1* in *Arabidopsis thaliana*. Interval targeted sequencing of the candidate region revealed a large fragment deletion in *Bo8g118320* in the glossy green cabbage Y2-1. In *B. napus*, Liu et al. [29] identified a lipid transfer protein gene, *BraLTP1*, that is involved in epicuticular wax deposition; overexpression of this gene in *B. napus* reduced wax deposition on leaves, with detailed wax analysis showing a 17–80% reduction in various major wax components. Liu et al. [30] identified that *BnA1.CER4* and *BnC1.CER4* cDNA are fatty acyl-coenzyme A reductase genes with a preference for branched substrates, which induce the accumulation of primary alcohols with chain lengths of 26 carbons. Wen et al. [31] identified a dominant GL and confirmed a drastic reduction in total wax content. Wax compositional analysis showed increased aldehydes but significant decreases in alkanes, ketones, and secondary alcohols. Ni et al. [32] reported that *BnUC1* is a key regulator of epidermal wax biosynthesis and lipid transport. Overexpression of *BnUC1^mut^* in ZS11 (Zhongshuang11) significantly reduced leaf epidermal wax content, resulting in curled-up and glossy leaves. In Chinese cabbage, Yang et al. [33] identified the *BrWAX2* gene, which is involved in epicuticular wax biosynthesis, through map-based cloning. They found that *BrWAX2*, in addition to reduced expression of other genes in the alkane-forming pathway, causes the glossy phenotype. Huo et al. [34] identified an AP2 transcription factor called *BrSHINE3*, which regulates wax accumulation in non-heading Chinese cabbage. Li et al. [35] discovered that an A BrLINE1-RUP insertion in *BrCER2* alters epicuticular wax biosynthesis. Song et al. [36] reported that a BrKCS6 mutation confers leaf brightness.

Together, these results suggest a series of genes involved in wax biosynthesis, including structural genes and transcription factors. Different genes may influence leaf wax formation through distinct metabolic pathways.

### 4.2. Wax Biosynthesis Pathway

Wax biosynthesis occurs in the endoplasmic reticulum [37]. First, C16 and C18 fatty acyl–acyl carrier proteins are hydrolyzed by acyl-ACP thioesterases and then converted into coenzyme A derivatives by long-chain acyl-CoA synthetases [10,38,39,40]. Second, the saturated C16 and C18 fatty acyl-CoA is subsequently transformed into very long-chain fatty acids by FA elongase complexes. This transformation is catalyzed by a series of enzymes: 3-ketoacyl-CoA synthase (KCS) mediates the formation of 3-ketoacyl-CoA, 3-ketoacyl-CoA reductase (KCR) reduces this intermediate to 3-hydroxyacyl-CoA, 3-hydroxyacyl-CoA dehydratase (HCD) dehydrates it to trans-2,3-enoyl-CoA, and trans-2,3-enoyl-CoA reductase (ECR) reduces the enoyl-CoA to a longer-chain fatty acyl-CoA [41,42,43,44,45,46]. Subsequently, very-long-chain fatty acids are converted into very-long-chain fatty acid-acyl-CoAs longer than C28 through the catalysis of CER2, with CER2-like proteins functioning together with CER6 [47,48,49]. Finally, the synthesized very-long-chain fatty acid-acyl-CoAs are transformed into aldehydes, alkanes, secondary alcohols, and ketones by CER1, CER3, and the CYTOCHROME B5 (CYTB5) complex [50,51,52,53]. In this study, the downregulated expressed genes in the α-linolenic acid metabolic pathway and the upregulated expressed genes (*ACSL*, *FAR*, *CER1* and *MAH1*) in the wax synthesis pathway in HQ2-1 jointly promote wax formation in HQ2-1 leaves.

In *B. napus*, Jin et al. [54] classified differentially expressed genes involved in cuticular wax biosynthesis according to their function in plant acyl lipid metabolism, including fatty acid-related pathways, wax and cutin biosynthesis pathways, and wax secretion. Wang et al. [18] identified a negatively regulated gene, *BnaC9.DEWAX1*, which is involved in wax biosynthesis via the transcriptional suppression of BnCER1-2 expression. In cabbage, Laila et al. [55] reported that the higher levels of expression of *LTP2* genes observed in a less waxy cabbage line and the higher level expression of the *CER3* gene in a more waxy line were likely associated with the lower and higher wax contents, respectively, in those two lines. These results suggest that both structural genes and transcription factors can regulate wax synthesis, although the regulatory mechanism of transcription factors involved in wax synthesis requires further exploration.

### 4.3. Regulation of Wax Biosynthesis in Response to Temperature

Environmental conditions such as drought, humidity, UV-B irradiation, extended darkness, and extreme salinity and temperature alter total wax accumulation and composition [56]. There is sufficient evidence linking cuticular wax biosynthesis to plant adaptability to temperature stress. Shephered and Griffths [56] reported that low temperatures (15–17 °C) or cold treatment (4 °C) significantly increased the total wax content in the leaves of *B. napus* and *Arabidopsis*. He et al. [57] found that *KCR1*, *LACS2*, and *ACC1* upregulation significantly increased very-long-chain fatty acids in T. salsuginea after 4 °C treatment. We also found that cold stress could significantly promote the biosynthesis of free fatty acids. Additionally, upregulation of genes in HQ2-1 under cold stress promoted α-linolenic acid metabolism pathway, indicating that more jasmonic acid may accumulate on the surface of HQ2-1 leaves under cold stress. These results suggest that cold-induced changes in the cuticle help protect plants from winter drought, although alterations in total wax accumulation and composition vary depending on the cold-treated plant species.

Our findings indicated that *BoCER1* controls leaf wax traits in *B. oleracea* and that upregulation of genes in HQ2-1 under cold stress promotes α-linolenic acid metabolism, thus promoting the accumulation of jasmonic acid on the surface of HQ2-1 leaves under cold stress and the accumulation of free fatty acids.

## 5. Conclusions

*BoCER1* was identified as a candidate gene for wax biosynthesis in the waxy cabbage HQ2-1. High levels of *BoCER1* expression promoted the downregulation of genes in the α-linolenic acid metabolic pathway and the upregulation of genes in the wax synthesis pathway in HQ2-1. Together, these changes contribute to wax formation in HQ2-1 leaves. Because the genes upregulated in HQ2-1 under cold stress promoted α-linolenic acid metabolic pathways, it is suspected that cold stress promotes the accumulation of jasmonic acid on the surface of HQ2-1 leaves. This study analyzed the gene expression changes involved in the formation of wax on cabbage leaves and confirmed that cold stress can induce the upregulation of α-linolenic acid metabolic pathway genes in cabbage.

## Figures and Tables

**Table 1:** Genetic analysis of wax-free trait phenotype.

Generation		Wax-Free	Waxy	Segregation Ratio	Expected Ratio	χ^2^
P1 × P2	F1-1	65	0	-	-	-
P2 × P1	F1-2	65	0	-	-	-
(P1 × P2) × P1	BC1-1	63	57	1.167:1	1:1	0.100
(P1 × P2) × P2	BC1-2	105	0	-	-	-
F2	F2	1191	384	3.102:1	3:1	0.072

## Data Availability

The dataset is available from the NCBI Short Read Archive (SRA) under the accession number PRJNA1370458.

## Supplementary Materials

The following supporting information can be downloaded at https://www.mdpi.com/article/10.3390/cimb48020152/s1.
