# Supplementary material for: Identification of Leaf Waxy Candidate Gene and Expression Changes in Related Genes in Response to Cold Stress of Cabbage (Brassica oleracea L.)
Source: Curr Issues Mol Biol. 2026 Jan 30;48(2):152. doi: 10.3390/cimb48020152 (PMC12939698; doi:10.3390/cimb48020152)
Supplement: Supplementary file 1 [file cimb-48-00152-s001.zip › supplementary figures.pdf]

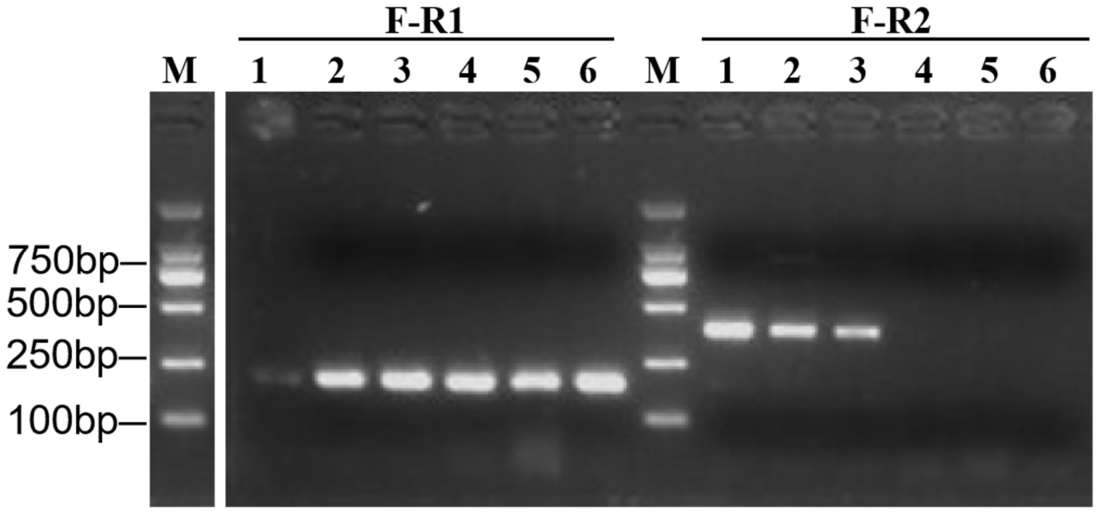

Figure S1. Functional dominant molecular marker design based on the sequence of *CER1*.

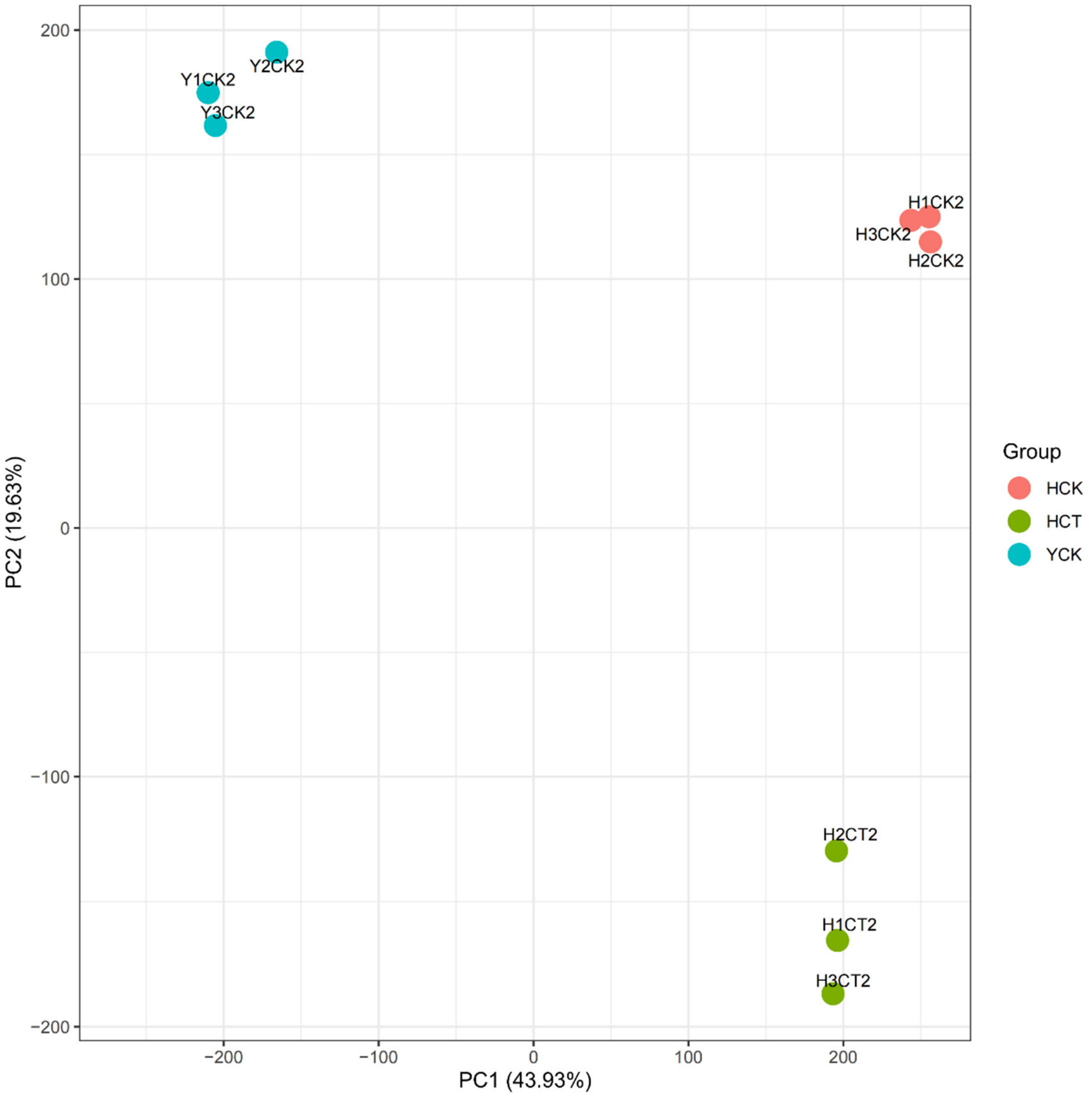

Figure S2. The principal component analysis diagram for samples.
