# Supplementary material for: Identification of Leaf Waxy Candidate Gene and Expression Changes in Related Genes in Response to Cold Stress of Cabbage (Brassica oleracea L.)
Source: Curr Issues Mol Biol. 2026 Jan 30;48(2):152. doi: 10.3390/cimb48020152 (PMC12939698; doi:10.3390/cimb48020152)
Supplement: Supplementary file 1 [file cimb-48-00152-s001.zip › supplementary table S1.pdf]

Supplementary table S1. Gene annotation information of 16 genes within the 182 kb genomic region.

No.	Gene	name	annotations
1	Bo8g117990		Sec14p-like phosphatidylinositol transfer family protein
2	Bo8g118020	TRM33	Phosphatidylinositol N-acetyglucosaminlytransferase subunit P-like protein
3	Bo8g118030		actin depolymerizing factor 11
4	Bo8g118050		propionyl-CoA carboxylase
5	Bo8g118070	PLIM2B	Encodes a member of the Arabidopsis LIM proteins
6	Bo8g118080		Encodes a member of the cation/proton antiporters-2 antiporter superfamily
7	Bo8g118140		SET domain-containing protein
8	Bo8g118160		Cyclophilin-like peptidyl-prolyl cis-trans isomerase family protein
9	Bo8g118170		Encodes a member of the armadillo/beta-catenin repeat kinesin motor family
10	Bo8g118190	STXBP1	syntaxin-binding protein 1
11	Bo8g118210	SPL8	Encodes an SBP-box gene, a member of the SPL gene family
12	Bo8g118240	CNOT1	CCR4-NOT transcription complex subunit 1
13	Bo8g118250	EREBP	EREBP-like factor
14	Bo8g118270	PPM2	tRNA wybutosine-synthesizing protein 4
15	Bo8g118310		-
16	Bo8g118320	CER1	Expression of the CER1 gene associated with production of stem epicuticular wax and pollen fertility
