# Supplementary material for: Identification of Leaf Waxy Candidate Gene and Expression Changes in Related Genes in Response to Cold Stress of Cabbage (Brassica oleracea L.)
Source: Curr Issues Mol Biol. 2026 Jan 30;48(2):152. doi: 10.3390/cimb48020152 (PMC12939698; doi:10.3390/cimb48020152)
Supplement: Supplementary file 1 [file cimb-48-00152-s001.zip › supplementary table S2.pdf]

Supplementary Table S2: Alignment analysis of RNA-seq with Brassica oleracea reference genome.

Sample	Total read pairs	Total mapped reads	Uniq mapped reads	Multiple mapped reads
Y1CK2	43243585	38353262 (88.69%)	36787133 (85.07%)	1566129 (3.62%)
Y2CK2	46578247	41345433 (88.77%)	39559878 (84.93%)	1785555 (3.83%)
Y3CK2	25825788	22465972 (86.99%)	21531462 (83.37%)	934510 (3.62%)
H1CK2	37369587	33118479 (88.62%)	31669553 (84.75%)	1448926 (3.88%)
H2CK2	42365474	37488047 (88.49%)	35878572 (84.69%)	1609475 (3.80%)
H3CK2	45405151	40204028 (88.55%)	38295863 (84.34%)	1908165 (4.20%)
H1CT2	32627066	28983054 (88.83%)	27857961 (85.38%)	1125093 (3.45%)
H2CT2	36507265	32446394 (88.88%)	31195026 (85.45%)	1251368 (3.43%)
H3CT2	21166540	18579326 (87.78%)	17092289 (80.75%)	1487037 (7.03%)
